# AI Chatbots and Cognitive Control: Enhancing Executive Functions Through Chatbot Interactions: A Systematic Review

**DOI:** 10.3390/brainsci15010047

**Published:** 2025-01-06

**Authors:** Pantelis Pergantis, Victoria Bamicha, Charalampos Skianis, Athanasios Drigas

**Affiliations:** 1 Net Media Lab & Mind & Brain R&D, Institute of Informatics & Telecommunications, National Centre of Scientific Research ‘Demokritos’, 153 41 Agia Paraskevi, Greece; vbamicha@iit.demokritos.gr; 2 Department of Information & Communication Systems Engineering, University of the Aegean, 832 00 Karlovasi, Greece; cskianis@aegean.gr

**Keywords:** executive function, artificial intelligence chatbot, conversational agents

## Abstract

**Background/Objectives**: The evolution of digital technology enhances the broadening of a person’s intellectual growth. Research points out that implementing innovative applications of the digital world improves human social, cognitive, and metacognitive behavior. Artificial intelligence chatbots are yet another innovative human-made construct. These are forms of software that simulate human conversation, understand and process user input, and provide personalized responses. Executive function includes a set of higher mental processes necessary for formulating, planning, and achieving a goal. The present study aims to investigate executive function reinforcement through artificial intelligence chatbots, outlining potentials, limitations, and future research suggestions. Specifically, the study examined three research questions: the use of conversational chatbots in executive functioning training, their impact on executive-cognitive skills, and the duration of any improvements. **Methods**: The assessment of the existing literature was implemented using the systematic review method, according to the PRISMA 2020 Principles. The avalanche search method was employed to conduct a source search in the following databases: Scopus, Web of Science, PubMed, and complementary Google Scholar. This systematic review included studies from 2021 to the present using experimental, observational, or mixed methods. It included studies using AI-based chatbots or conversationalists to support executive functions, such as anxiety, stress, depression, memory, attention, cognitive load, and behavioral changes. In addition, this study included general populations with specific neurological conditions, all peer-reviewed, written in English, and with full-text access. However, the study excluded studies before 2021, the literature reviews, systematic reviews, non-AI-based chatbots or conversationalists, studies not targeting the range of executive skills and abilities, studies not written in English, and studies without open access. The criteria aligned with the study objectives, ensuring a focus on AI chatbots and the impact of conversational agents on executive function. The initial collection totaled *n* = 115 articles; however, the eligibility requirements led to the final selection of *n* = 10 studies. **Results**: The findings of the studies suggested positive effects of using AI chatbots to enhance and improve executive skills. Although, several limitations were identified, making it still difficult to generalize and reproduce their effects. **Conclusions**: AI chatbots are an innovative artificial intelligence tool that can function as a digital assistant for learning and expanding executive skills, contributing to the cognitive, metacognitive, and social development of the individual. However, its use in executive skills training is at a primary stage. The findings highlighted the need for a unified framework for reference and future studies, better study designs, diverse populations, larger sample sizes of participants, and longitudinal studies that observe the long-term effects of their use.

## 1. Introduction

The transition from Industry 3.0 to Industry 4.0 focuses on achieving digitalization and creating a virtual framework that combines the coexistence of digital, physical, and biological systems. However, the successful integration of Industry 4.0 requires the growth of adaptive and analytical thinking skills directly linked to information technology and data analysis. Consequently, there is a need for personalized skills training and the necessary know-how in the workforce to respond to the new conditions [1].

Artificial intelligence (AI) is one of the most fundamental technologies of the future, creating intelligent machines and AI systems that process problems and complex data. The continuous and progressive changes in its field and its integration into human society make it essential for flexibility to adapt to new data, facilitating our everyday lives [2].

The evolution of technology and information technology has highlighted the use of electronic chatbots, the conversational agents (CAs), in the service of various fields of science and entrepreneurship. In the literature, we find the terms chatbots, conversational artificial intelligence, artificial intelligence chatbots, and conversational agents used interchangeably [3]. Chatbots, taking on different roles, act in a supportive manner towards humans, enhancing their intelligence through verbal ability and the interaction that develops [4]. Their application in various fields is becoming increasingly popular. Specifically, by 2025, their global market is expected to grow to almost $1.23 billion [5].

The chatbot talks and interacts with the human agent through spoken, written, and visual languages. It can mimic human behavior and execute specific tasks, intelligently conversing with users. Lately, because of the increasing advancements in artificial intelligence and machine learning, chatbots seem to be increasingly used in financial services, customer service, education, and healthcare. Some of them can have their personality, processing and storing information from the context of their interaction with the user, giving more accurate answers. Moreover, by gradually acquiring more information about the user, they learn it better and perform better to the demands of the conditions [6].

Executive Functioning (EF) plays an essential role in human learning and performance by aiding in the management and regulation of thought and action. Executive skills are predictive factors for the child’s success in the school environment, academic course, and well-being as an adult in every area of his life. In particular, they constitute a set of mental and problem-solving abilities to achieve a future goal [7].

The mental processes of the executive mechanism allow the ability to maintain information in working memory, inhibit immediate responses to stimuli, and shift attention between distinct aspects of a task or problem. In addition, they provide control over our behavior, inhibit unwanted actions and behaviors, focus attention, and organize our thinking. Significantly, they tend to improve the greater we practice them but are limited by the stress and pressure the individual experiences in everyday life, including genetic, neurobiological, and environmental factors [8,9,10].

Plenty of research conducted throughout the past few decades demonstrates the role of executive functions in the individual’s performance in familiar challenges. For example, updating the contents of working memory enhances dual-tasking, goal focus, logical reasoning, and planning [11].

AI chatbot integration in the educational process, mainly in higher education, combines educational innovation with the development of higher cognitive abilities related to problem-solving, critical thinking, self-regulation, and metacognition [12]. Mitsea et al. [13] point out that AI chatbots may serve as tools supporting training, guidance, and user feedback on newly acquired abilities.

However, research investigating the contribution of such tools to the development of executive skills is limited. Studies focus more on utilizing artificial systems in the health sector and clinical populations. In an educational context, learners of different ages rely on multiple cognitive processes for goal-directed behavior, with the executive mechanism playing a prominent role in their long-term academic success. This area of study linking applying AI chatbots to enhancing executive skills training is at a preliminary stage. But, considering that it reinforces the traditional way of learning, maintaining great accessibility and autonomy with the possibility of enriching cognitive processes, it signals a need for further research [14].

Some studies drawing research interest from the possibility of enhancing or training executive skills through AI chatbots presented their findings. The research by Chauncey and McKenna [15] explored the programmability of AI Chatbots to generate responses using multiple knowledge search paths and contexts, allowing the user to gain a deeper understanding of a specific topic. Specifically, they implemented an iterative process of asking a core question to the AI chatbot (ChatGPT-3.5) and then asking the same question from various perspectives and with specific boundaries. Boundary-setting and perspective-taking were employed to contribute to a broader understanding of a problem and approach a topic from different perspectives. As a result, researchers report that large language model artificial intelligence (AI) chatbots can enhance users’ cognitive flexibility by reducing cognitive rigidity. A Large Language Model (LLM) is a complex model trained on a large amount of data that produces language that simulates human speech. Some of these models are GPT, PaLM, Jurassic, and Claude [16,17].

In a later study, Chauncey and McKenna [16] investigated whether utilizing large language model AI chatbots can enhance cognitive flexibility alongside creativity and innovation in complex urban digital environments. Their research emphasizes that such artificial systems assist in exploring problems, enabling timely planning and evaluating responses. Additionally, the interaction between a human agent and a chatbot fosters cognitive flexibility and ongoing engagement, promoting the generation of creative ideas and their implementation in innovative actions.

A recent study by Klarin et al. [14] focused on the relationship of applying LLM artificial intelligence chatbots in schoolwork to adolescent executive function, including planning, inhibition, and cognitive flexibility. This research is the first attempt to examine these parameters among teenagers. The results indicate that adolescents with limited EF benefit from using AI chatbots in task completion.

Chauncey and McKenna [17] emphasize that cognitive flexibility is developed gradually by human interaction with the LLM AI Chatbot. The chatbot’s instant response replay feature promotes critical thinking by evaluating responses. Consequently, the cognitive load on the user is limited, causing self-regulation in their attention, behavior, and overall action. Its cognitive flexibility then emerges, encouraging information-seeking and engagement behaviors during the inquiry phase of learning. This engagement leads to alternatives that promote reflection, cognitive flexibility, understanding, and problem-solving.

The approach of Saengrith et al. [18] leveraged the use of a blended learning model through chatbots to enhance problem-solving skills in the workplace. The online training platform LINE chatbot guided learners to achieve their goals, improving learning through the interactive real-time response feature. Parsakia [19] asserts that chatbots can enhance problem-solving skills by accelerating learning and contributing to higher cognitive tasks. In particular, artificial systems promote interactive learning experiences with structured guidance and activate skills related to problem-solving, such as logical reasoning, hypothesis testing, and decision-making. However, it is necessary through these processes to strengthen independent thinking and the possibility of adaptation and alternative actions in complex, unexpected conditions.

The essential research of Sáiz-Manzanares et al. [20] points out that chatbot applications integrated into virtual learning spaces seem profitable for people with special educational needs, such as vision problems or symptoms of attention deficit. Artificial systems encourage learners to reflect on their thinking about what is troubling them. In addition, the users’ processing of their responses prompts them to engage in metacognitive strategies, enhancing self-regulation and autonomy in problem-solving and decision-making. A previous study by Jang et al. 2021 [21] examined the effect of a 4-week intervention of an interactive mobile-based chatbot application in individuals with attention deficit symptoms. They used a digital therapy tool that provided easy access and self-guided management. The user could follow self-education programs through predefined dialogs. It has psycho-educational and self-educational functions that evolve incrementally at different levels. The research results showed an improvement in the intensity of attention deficit symptoms, with positive effects on concentration, emotional self-regulation, inattention, memory capacity, and empathy. Another study [22] found similar findings regarding chatbots’ usage for schoolwork and their influence on students’ working memory. Chatbots helped reduce cognitive load, contributing to student efficiency and productivity. In addition, they emotionally strengthened the trainees, reducing stress. Notably, artificial systems applications reduce the increased mental effort in various tasks, preventing cognitive fatigue and promoting performance and attention in working memory.

Several studies report the contribution of chatbots to the enhancement of self-observation and the control of emotions and memory. At the same time, the user develops his metacognitive judgment, evaluating the correctness of his actions. In particular, a chatbot can lead the user to cognitive change, guiding them to self-examination and reflection. The human agent hypothesizes, analyzes the consequences of his actions, and improves his self-understanding. Specifically, creating new connections between the information available to the user enables cognitive restructuring [23].

Karyotaki et al. [24] presented an innovative e-learning tool. It is an original conversational agent that aims to develop cognitive and metacognitive skills, promoting the management of conditions of healthy aging and sustainable living. The AI chatbot combines supervised machine learning algorithms and augmented intelligence and is usable as a personal tutor and training assistant. The artificial factor focuses on self-regulation, the control of attention as a metacognitive skill, directly influencing human behavior. The AI chatbot enhances quality education through innovative learning environments and empowers teachers through applications that reflect an equal, unrestricted education.

The application of technology tools, including AI chatbots, has many advantages that are decisive in their rapid development. Nevertheless, their use has some risks and challenges due to their malicious management or the promotion of inequalities in some cases. It is essential to consider parameters like transparency, accuracy, reliability, security, privacy, and accessibility features in their design and programming [17].

The current study aims to explore opportunities for enhancing and training executive skills through AI chatbots by utilizing the systematic review method based on the PRISMA 2020 Principles. The following databases were used for the source search: PubMed, Web of Science, Scopus, and Google Scholar, using the snowball search method. The study focused on three specific research questions: the role of conversational chatbots in training executive functioning, their effect on executive-cognitive skills, and the duration of any observed improvements. Out of the *n* = 115 publications initially collected, only *n* = 10 were selected due to carefully defined qualifying criteria, ensuring a focused and high-quality research outcome. The research results reflect the possibility of improving and expanding executive skills, emphasizing working memory, attention, self-regulation, cognitive flexibility, problem-solving, decision-making, and metacognitive skills from the controlled use of AI chatbots. In addition, the promotion of adaptive capacity, reflection, autonomy, critical thinking, and the reduction of anxiety creates positive emotions, which indirectly enhance the emotional development of the user with an impact on his behavior. The study illustrates how AI chatbots are still in their infancy as a tool for teaching higher-order mental skills, suggesting the future possibilities of their efficient use in executive and cognitive mechanisms growth. Thus, it may catalyze additional study in education and the responsible application of artificial intelligence, focusing on the optimal development of specialized usage of AI chatbots that enhance executive function.

More specifically, this study is groundbreaking, as it is the first to comprehensively explore the potential and numerous benefits of using AI chatbots as therapeutic agents for the executive mechanism. It shows how these innovative tools can effectively improve aspects of executive functions, ultimately paving the way for improved mental health and well-being. However, the study findings highlighted significant restrictions, such as unobserved direct effects, small sample sizes, lack of diverse populations, controlled settings that do not generalize to real-world situations, and limited diversity.

Section 2 follows the introduction, which addresses the study’s methodology, and then Section 3 approaches the theoretical knowledge, including the role of artificial intelligence chatbots in education and therapy as well as their impact on enhancing executive skills. The study concludes with Section 4, Section 5 and Section 6, which present the results, discussion, and conclusions.

## 2. Materials and Methods

### 2.1. Study Design

This study design applied a systematic review approach of the literature using the PRISMA statement for systematic reviews and meta-analyses following the process of identification, selection, assessment, and synthesis of the included studies reassuring the following methodological steps that were performed. Open Science Framework was used upon registration of the systematic review protocol (accessed on 7 December 2024 https://osf.io/jxmg4). This systematic review attempts to answer the following research questions for the final selected studies that were analyzed.

RQ1.What types of conversational chatbots were used for improving executive functions?
RQ2. What were their outcomes in executive functions?
RQ3. What was their duration of the effects in executive functions?

### 2.2. Inclusion-Exclusion Criteria

The following inclusion and exclusion criteria were applied to this systematic review based on our objective and research questions.

IC1. Inclusion of studies performed from 2021 to the present.
IC2. Be experimental, observational, or both, including quantitative, qualitative, or mixed methods.
IC3. Inclusion of studies that have specifically used AI-based chatbots or conversational agents to support executive functions or specific conditions that affect them in the form of stress, anxiety, depression, memory, attention, cognitive load, and behavioral changes.
IC.4 Inclusion of studies with both general or populations with specific neurodevelopmental or neurological conditions.
IC.5 All studies included were peer-reviewed.
IC.6. All studies were written in the English language.
IC.7 All studies included had full-text access for the full data extraction process.

On the other hand, the exclusion criteria were the following:

EC1. Studies before 2021 were excluded.
EC.2 The literature reviews, systematic reviews and metanalysis were excluded.
EC.3 Every study that included non-AI-based chatbots or conversational agents like mobile applications, non-interactive digital tools, or exclusively human-performed therapies was excluded.
EC.4 Studies that did not target the EF’s range of skills and abilities were excluded.
EC.5 Studies that were not written in the English language were excluded.
EC.6 Studies that were not open access.
The following criteria are aligned with the study’s objectives, reassuring the focus on AI chatbots and conversational agents on the impact they have on EFs.

### 2.3. Databases Screened and Selection Process

The following databases were used to systematically analyze the literature. Web of Science, Scopus, PubMed, and complementary Google Scholar were used to provide deeper analysis as well as peer-reviewed and open-access articles. The methodology performed was based on PRISMA 2020 guidelines, also utilizing the method of snowball research. The following keywords were utilized to provide the most results: “AI chatbots”, “chatbots”, “conversational agents”, “executive functions”, “attention”, “memory”, “cognitive load”, and “emotional regulation”. The first stage was to outline the requirements for eligibility (Table 1).

The databases’ filters and Boolean operators were then used in an advanced search. We began additional processing as soon as the candidate studies were chosen. Priority was given to studies that contained the predetermined terms in both the abstract and the title during the abstract/title screening step. References that did not fit the eligibility requirements were then excluded, and duplicate papers were eliminated. The software Mendeley Desktop 1.19.8 was used to exclude redundant references. The full-text screening stage involved additional processing of the remaining studies. At this point, the full-text documents were obtained, and the content was thoroughly examined. Other criteria, like the methodological procedures followed in each study, were also taken into consideration while making the selection. The eligible studies were independently determined by two reviewers (P.P) and (V.B). Accepted, refused, or undecided were the options available to the reviewers. With the third (C.S) and fourth (A.D) reviewer’s input, a discussion ensued in case the other reviewers were unclear or disagreed. Following the evaluation of the research, the reviewers gathered the pertinent data and arranged the information in tables. The findings were then synthesized. A critical overview, analysis, and evaluation of the body of evidence covered in this review were provided by the qualitative synthesis.

### 2.4. Data Extraction

Aspects of the studies’ structure (study design, aim, conditions, variables/measure, key findings), participant demographics, information (authors, year, country), and specific information pertaining to the research questions formulated were among the data taken from the included studies.

### 2.5. Included Studies

Following processing, inclusion, and representative criteria for the main part (*n* = 10) in the final selection for additional research and analysis, a total of *n* = 115 articles were found and screened for the final selection of the included articles. The literature review was conducted from January 2021 to 2024.

Eligibility criteria were established as the initial stage of the selection procedure. Furthermore, a list of keywords was created to start the database search, employing a variety of search filters and Boolean operators to obtain the biggest number of results. Following the elimination of duplicate studies (*n* = 8), the selection process was conducted using title and abstract screening following the eligibility criteria (*n* = 115).

**Figure 1:**
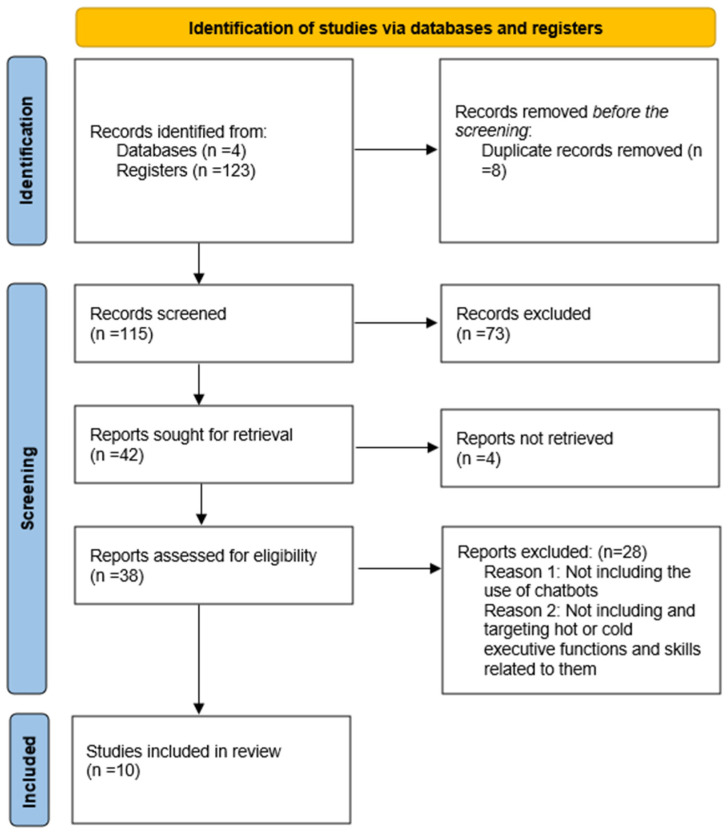
Prisma 2020 chart flow.

Following the exclusion of (*n* = 73) publications, the full-text screening was the next step in the procedure. A thorough processing was performed on the remaining studies (*n* = 42). Unfortunately, it was not possible to retrieve *n* = 4 articles for full-text screening. Two independent reviewers took part in the final selection process, examining the entire texts of (*n* = 38) papers and debating whether the eligibility requirements were applicable. Because they did not meet the eligibility requirements, the remaining (*n* = 28) were eliminated. As a result, the final selection included *n* = 10 items (Figure 1).

### 2.6. Studies’ Characteristics

**Figure 2:**
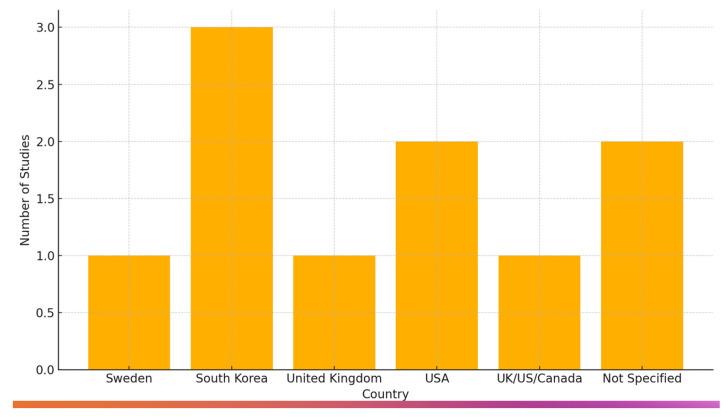
Distribution of studies by country.

**Figure 3:**
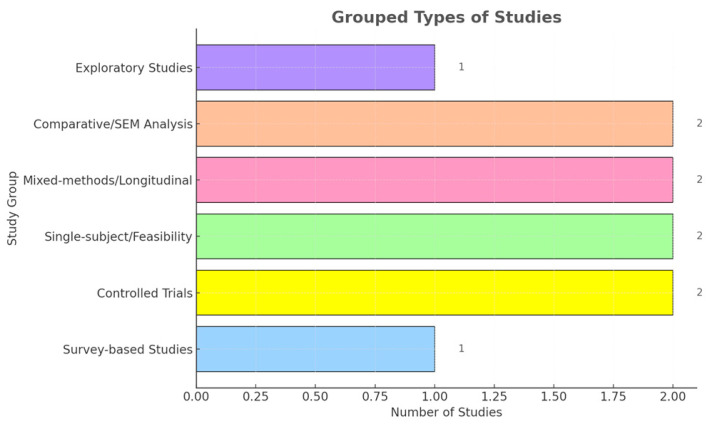
Methodology distribution in studies.

From the final study selection, three distinct categories were derived according to the age groups of participants. These included children and adolescents, university students and young adults, adults, and older adults. A total of *n* = 1606 participants were analyzed due to their interaction and the effect of AI chatbots on their higher mental abilities and condition. More specifically, we collected data for the ages 6–75+ years of age. According to the country of each study, we observe a diverse variety, more specifically *n* = 3 [21,25,26] from Republic of Korea, *n* = 2 from the USA [27,28], *n* = 1 from Sweden [14], *n* = 1 from the UK [22], and *n* = 1 of them were multinational, including countries like the USA, UK, and Canada [29], unfortunately, *n* = 2 [30,31] of the included studies did not specify the exact location of the participants. According to the study design, the researchers established several different kinds of methodologies. These include randomized control studies, quasi-experimental designs, feasibility studies, single-subject designs, as well as observational studies. Others established mixed methods offering both quantitative and qualitative data to support their research objectives (Figure 2 and Figure 3).

## 3. Theoretical Background

The following section includes three subsections. The first two are related to the theoretical background of concepts concerning executive functions and Artificial Intelligence systems, such as chatbots. The third and final subsections describe the role of chatbots in education.

### 3.1. Executive Function

Cognitive, developmental psychology, neuropsychology, and education studies have explored the cognitive domain of executive function, a multidimensional construct with developmental and individual differences. In many cases, the direct measurement of these processes is challenging due to the complexity and assessment tasks [7].

Executive skills like working memory, cognitive flexibility, and inhibiting unrelated thoughts are crucial for achieving goals and essential for human abilities and achievements. However, deficiencies in these skills can lead to neurological disorders [32].

Pergantis et al. [9] highlight the importance of basic executive skills, which require cooperation and interaction to achieve goals.

Self-regulation is a complex process that involves observing, planning, controlling, and adjusting one’s behavior to accomplish objectives and maintain focus [33].

Attention plays a vital role in executive function by allowing us to monitor and focus on tasks effectively. It also helps filter information for storage and processing in working memory [7,34]. We distinguish five levels of attention: focused, sustained, selective, alternating, and divided, each with its respective interventions. Focused attention is intentional, sustained attention is prolonged, selective attention ignores other stimuli, and alternating attention shifts between stimuli [35].

Cognitive flexibility involves mental representation and vigilance, allowing individuals to perceive multiple perspectives and quickly adapt to changes in their thinking [15].

Planning is a mental process that involves formulating, evaluating, and selecting actions to achieve a goal [36]. Problem-solving is a high-level cognitive process that involves coordinating other cognitive functions like perception, attention, and memory and regulating cognitive and social factors [37].

Working memory comprises a set of structures and processes that allow temporary storage, processing, and manipulation of information. It is directly related to the ability to pay attention, which can affect the capacity and performance of working memory [38].

Inhibitory control refers to attention control, thoughts, emotions, and behavior, bypassing unnecessary processes. Consequently, it creates opportunities for precise reactions and adaptability. [39]. Reasoning involves generalization and abstract thinking skills, promoting concept generation, creativity, and innovation [36].

Using executive skills stimulates the brain’s neural networks in the prefrontal cortex, which includes several areas. The limbic system and the prefrontal cortex are connected, with neurotransmitters such as dopamine, norepinephrine, and cortisol that regulate and enhance neural activity in the prefrontal cortex (PFC), positively influencing executive functions [8].

Moreover, executive functions are divided into “hot” and “cool” dimensions: emotional and cognitive control. Cold executive functions involve logical analysis and planning, while hot emotional aspects focus on individual motivations, empathy, self-awareness, and decision-making related to emotional states [7,33].

Problem and situation management combines warm and cold executive function skills to solve problems or pursue goals. Educational literature links executive function to metacognition and self-regulated learning, with individual skills improving through experience and practice [7,39]. According to research, executive functioning can be trained and improved at various ages through repetition in different practices. Notably, enhancing practice is linked to its effectiveness in managing and overcoming challenges [9,40].

### 3.2. Artificial Intelligence (AI) Chatbots

Chatbots are computer programs that interact with the user automatically, leveraging text- or speech-based dialog. Users develop a conversation with a technical system through interactive contact, allowing users access to functions and data of the application itself. Chatbots are used in customer communication in e-shopping, teaching, mental health promotion, the game industry, and especially in the financial sector [2]. It is worth noting that chatbot design aims to facilitate humans in their work and their interaction with computers, using natural language, without undermining the human factor [41].

Natural Language Processing (NLP), a Dialog Manager, and content are the three primary components of a chatbot. NLP is a component of artificial intelligence that enables human-chatbot interaction, in which the user communicates with it using a programming language. It uses natural language processing through syntactic and semantic analysis. Dialog Manager selects the message the user will receive based on their previous interactions. What the artificial system transmits for human perception is called content [42].

Chatbots offer a corpus of knowledge that defines their field of expertise. Generic chatbots answer user questions from any domain, while cross chatbots cover multiple domains. Interpersonal chatbots provide services, while intrapersonal chatbots live in users’ spaces and understand their needs. Inter-agent chatbots communicate with other chatbots [43].

Chatbots are advanced tools that accurately interpret human abilities and user needs, providing immediate and accurate responses. Moreover, chatbots are human-created brainpower programs that can shape and direct conversations. Their effectiveness is related to the involvement of Text Classifiers, Counterfeit Neural, Systems Appropriate Calculations use, and Characteristic Language preparation [44]. Traditional rule-based chatbot models face challenges with non-predefined questions, while retrieval-based models match user queries but have their limitations. Generative models address these weaknesses through natural language processing (NLP) and deep learning techniques to model and train the chatbot system [45].

AI chatbots leverage machine learning for human cognitive functions such as problem-solving and decision-making. In addition, they can understand the user’s intentions and feelings, enhancing their communication. A helpful tool in this process is natural language processing (NLP), which has been evolving significantly in recent years, aiding in understanding tasks and creating higher-level contexts [3].

Researchers developed Context-Aware Chatbots (CACs) with human-like features, enhancing natural interaction. Personality-Aware Chatbots (PACs) were categorized into Self-Personality-Aware Chatbots (SPAC) and Other-Personality-Aware Chatbots (OPAC). SPACs have predefined personality traits, while OPACs can adapt their behavior and responses to fit the user’s personality. More specifically, chatbots can enhance user experiences and cognitive-emotional information by modifying their behavior, providing personalized responses, and adapting to the user’s personality traits [45].

### 3.3. Chatbot’s Role in the Educational Process: Restrictions Arising from Their Use

The context of Industry 4.0 shapes a constantly changing technological landscape that pushes to redefine the traditional educational character, developing a mindset of continuous learning. Artificial intelligence tools significantly influence scientific and social fields through their involvement in educational processes. A case in point is that AI chatbots have revolutionized education by offering personalized actions, autonomy, and interactive engagement [1].

Digital transformation is a crucial aspect of the development of various academic disciplines. Introducing new technologies, skills, and innovative organizational models highlights new work perspectives, combining creative solutions. At the base of the new conditions is the field of education at all levels. Pupils and students are asked to adapt to the recently developed educational environments, effectively involving physical and virtual learning strategies and collecting data and knowledge without time or location limitations. A new form of educational process called Education 4.0 is gradually taking shape [46].

Education 4.0 presupposes the creation of new educational contexts and environments. The new digital world involves transformations in modern education, including flipped classrooms, blended learning, self-regulated learning, project-based learning, inquiry-based pedagogy, and digital tools at all educational levels [47].

Integrating artificial intelligence into our daily lives requires distinguishing between weak AI and Artificial General Intelligence (AGI). Weak AI refers to computer programs that aim to solve specific problems using AI techniques such as data mining and machine learning. In contrast, AGI leverages flexible artificial systems that solve problems, approximating human action [48].

The application of generative artificial intelligence in education is an emerging field in educational technology. AI applications are reshaping teaching and learning by providing instant feedback, assessment, and personalized instruction, thereby enhancing the provision of resources and learning materials to meet user needs. AI tools like chatbots enhance the educational process, necessitating human guidance and evaluation of their results. Their design and implementation can respond to an inclusive education, as it can compensate for issues of integration and equality in the educational sector. However, it is imperative to provide training and support to teachers to maximize their application potential and ensure their ethical use [49].

Chatbots have been used for several years as teaching agents in educational environments, with positive learning results. Importantly, they have no time or location boundaries, as they can enhance the learning process from anywhere at any time [50].

AI chatbot is emerging as an effective tool that can act as a virtual assistant, giving learners the ability to interact, ask, and gain experience from anywhere. Essentially, the artificial agent offers support to users according to their needs, goals, and levels of self-awareness, creating possibilities for the development of their self-learning [51].

Chatbots in education can improve student learning, understanding, cognitive performance, and real-time observation of trainee effectiveness, catering to diverse learning needs and interests [52].

Educational chatbots, Pedagogical Conversational Agents (PCA), and intelligent software agents of the Internet of Things (IoT) can enhance adaptive learning and skill acquisition [53]. The Socratic method is highly effective in chatbots for enhancing higher-order cognitive abilities. The dialectical technique used, through stimulating responses, promotes the user’s critical thinking and self-analysis [54]. In addition, chatbots utilize cognitive and emotional feedback types to provide support and positively impact the emotional state. An example of emotional dialog is the indicator, where phrases of encouragement are used to provide positive reinforcement to users. Both cognitive and affective feedback from chatbots significantly enhance metacognitive reasoning and self-regulation [53].

Yin et al. [55] highlight the significant role of emotions in learning events, influencing cognitive processes like attention, working memory, and information processing. Positive emotions facilitate information retention and improve problem-solving abilities. Negative emotions, such as anxiety, significantly impact memory, information management, and mental task performance. Furthermore, Yetişensoy and Karaduman’s study [56] highlights chatbots for personalized, autonomous learning, contributing to social development education through data-driven experiences.

Artificial intelligence research has considerably improved large language models (LLMs) that simulate human intelligence by helping users discover different and new perspectives. In addition, they facilitate and enhance decision-making through information, creating engaging experiences that can support cognitive processes in real-world scenarios [57].

AI chatbots powered by LLMs possess distinctive capabilities due to extensive training datasets and advancements in natural language processing (NLP). In addition, they show emergent abilities in reasoning, planning, decision-making, and learning in context due to the sheer scale of their trained material [14]. Intelligent artificial systems, as new artificial intelligence technologies, are gradually gaining an essential role in the formation of innovative teaching and learning processes and methods. As a result, teachers are adapting their thinking and actions to the evolving educational system in the context of Education 4.0 [58].

The traditional education system can be defined by overcrowded classrooms, limited personalized instruction, varying learning rates, and the need to adapt to rapid technological and information advancements. Under these conditions, the gradual rise and implementation of AI chatbots emerge as a promising solution to these issues. Educational institutions are gradually integrating artificial systems into their learning environments, assessing their potential benefits and threats. Perhaps in the upcoming years, the use of chatbots in education is expected to significantly improve the educational process and learning experience [59].

Educational institutions at various levels of education and professional development programs use AI chatbots. Personalized education is a crucial aspect of excellence in fostering critical and analytical thinking in individuals. However, research reports that they provide savings and efficiency, autonomy, and flexibility in learning [60].

Sandu and Gide [48] emphasize the benefits that education can gain from chatbots. They point to enhancing productivity, communication, learning, teaching aids, and reducing ambiguity in interaction. Furthermore, they mention the important distinction between traditional teacher-centered and student-centered educational systems, incorporating AI in the context of Education 4.0.

Klarin et al. [14] point out a gradual rise in the utilization of generative AI tools in higher education. University students use AI chatbots in tasks related to text analysis, writing texts, problem-solving, structuring presentations, decision-making, and social support. Research results indirectly show that the e-application of AI tools can enhance behaviors that require the EF involved in various cognitive processes. Therefore, skills such as attention, working memory, inhibition, planning, cognitive flexibility, observation, and concentration coexist and interact in cognitive environments to achieve tasks that can be enhanced by focused training mediation of AI chatbots and demand mental effort.

Many studies outline the benefits of using chatbots in education and academia, pointing to improved research efficiency and accuracy. The process involves swiftly analyzing vast amounts of data and identifying patterns and linkages that are not easily discernible by humans due to their complexity [61]. However, some of the risks and limitations that chatbots present are the violation of privacy, the violation of intellectual property, the erosion of academic integrity, the accuracy of the information they provide, and the limited interaction with instructors and other people [12]. Chatbot systems utilize AI to enhance user control over their data and improve the accuracy and user experience during interactions. An illustrative example is chatbots with NLP applications that enable the creation of asynchronous follow-up questions and use neural networks for emotion detection in conversations [62].

However, the critical evaluation of the ethical and technical consequences of their e-application by humans is necessary to ensure their responsible and transparent use. Furthermore, their role fosters human expertise, judgment, creativity, and flexible problem-solving. The continuous development of technology and the increasing presence of AI in our lives create the need to adapt to new conditions. Intelligent systems are gradually entering the field of the educational environment, essentially contributing to the training of cognitive and social skills [61].

However, AI implementation in education raises ethical concerns regarding privacy, bias, transparency, and accessibility due to worries over AI algorithms. Moreover, significant restrictions, such as their careless use and the misleading or false information they may display, relate to their data integration [42]. The literature describes the erroneous results of AI chatbots as hallucinations or AI artifacts attributed to a misalignment between user expectations and the capabilities of the chatbot AI [63,64]. The inherent limitations of Generative AI, such as inconsistent responses and contradictory responses, can undermine its reliability and reduce user trust [65].

Some developers propose encoding/decoding transformer malfunctions or manipulating bibliographic data to address the problem. Consequently, examining hallucinations and their consequences in AI chatbots is crucial for the effective use of artificial intelligence in various fields [63]. Hence, there is a demand for awareness, adoption of legislation, and consolidation of moral values so that chatbots and all AI tools can serve as catalysts for additional growth and innovation [26].

Coordinated cognitive and social human-artificial system interaction with an anthropocentric role involving ethical demarcation and necessary scientific constraints may enhance mutual communication, social cognition, cognitive development, and collaborative action [66].

**Figure 4:**
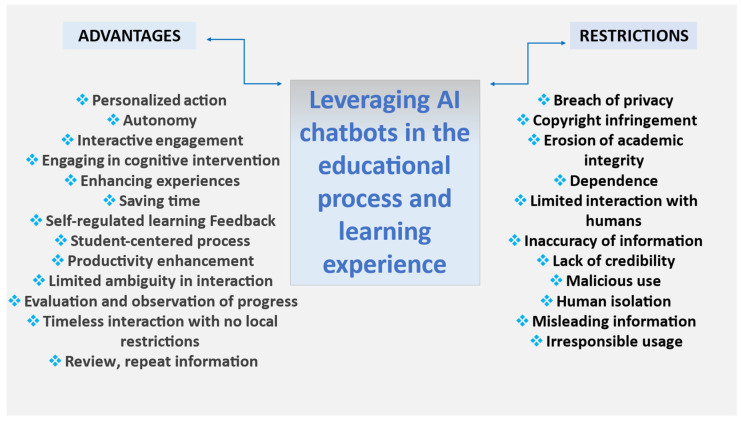
AI chatbot utilization in educational interaction. Benefits and limitations.

Summarizing the findings of various studies [1,3,12,21,48,55,56,60], the application of AI chatbots in the educational process is beneficial, with dominant elements of autonomy, specialized learning, self-learning, and feedback. However, their use involves risks and ethical limitations regarding their design, programming, and reckless and malicious use (Figure 4).

## 4. Results

### 4.1. RQ1 What Types of Conversational Chatbots Were Used?

Mauriello et al. 2021 [27] used a decision tree chatbot (also known as Popbots) delivering micro-interventions in *n* = 47 adults including *n* = 14 students and *n* = 33 staff aged 18–74 years of age, for the measurement processes daily surveys assessed stress Patient Health Questionnaire (PHQ-4) and engagement metrics (frequency and duration of chatbot use). Furthermore, Klarin et al., 2024 [14] also utilized Generative AI chatbots (e.g., ChatGPT) in 744 adolescents aged 12–19 years in their studies (Study 1: 385, Study 2: 359), while assessing chatbot use, perceived usefulness, and executive functioning utilizing BRIEF-2 and through analysis of academic grades. Another study by Fabio et al., 2024 [31] used general-purpose AI in their study of 126 university students aged 18–32 with ANOVA/MANOVA testing cognitive reasoning, openness, and fluency via standardized critical thinking tasks. Koivisto and Grassini, 2023 [29] also used Generative AI (ChatGPT-3.5/4, Copy.ai) for 256 participants aged 19–40 from English-speaking countries. The measurement was performed through semantic distance metrics and subjective creativity ratings to compare human and AI performance in Alternate Uses Tasks (AUT). Finally, Rostami and Abadi, 2023 [22], employed Generative AI in 3 students aged 11–13 years of age utilizing the N-Back test to assess working memory before/after chatbot use in schoolwork.

**Figure 5:**
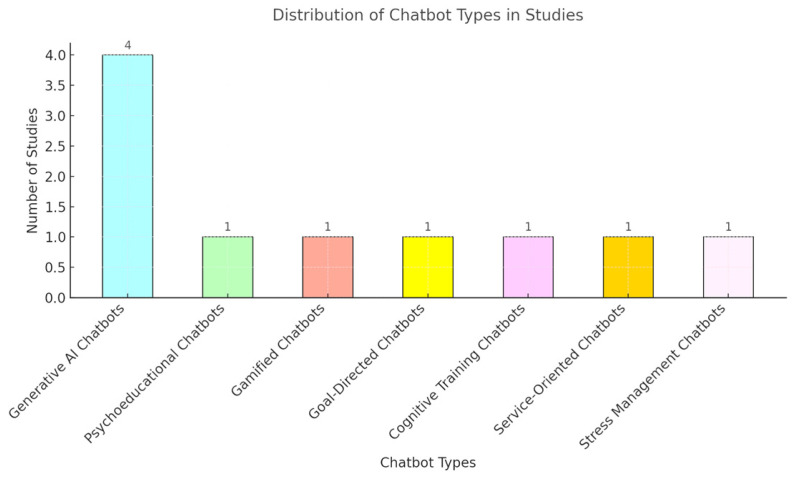
Distribution of types of chatbots used in studies.

Other researchers operated specified or mixed gamified chatbots to enable their target groups’ cognitive functions. Park et al., 2024 [26] used a goal-directed chatbot (ForME) in 132 children aged 6–7 with ADHD symptoms using measures of ADHD RS and executive function (BRIEF), which was assessed via parent/teacher feedback and chatbot adherence logs. Kim et al., 2024 [28] also applied a type of cognitive training chatbot in *n* = 32 older adults aged 60+ years of age utilizing memory tasks assessed with CANTAB (episodic/working memory) evaluating mental health, using GDS and GAI scales. Jang et al., 2021 [21] used a psychoeducational chatbot delivering CBT in 46 adults with ADHD aged 19–60 years of age with ADHD symptoms utilizing measurements through (CAARS), anxiety (SAS), and depression (QIDS-SR), which were performed pre/post-intervention. Sun et al., 2023 [25] in their study with the gamified chatbot (NUROW) targeting cognitive skills, evaluated in 27 children aged 6–12 years of age with ADHD symptoms (ARS), behavioral outcomes (CBCL), and neuropsychological tests measuring cognitive improvement. Finally, Chou and Hsu, 2021 [30] established a service-oriented chatbot for cognitive load management in 193 chatbot users, performing SEM analysis of system and information quality impact on user cognitive load, attitudes, and usage intentions (Figure 5).

### 4.2. RQ2. What Was the Outcome of Executive-Cognitive Functions?

**Figure 6:**
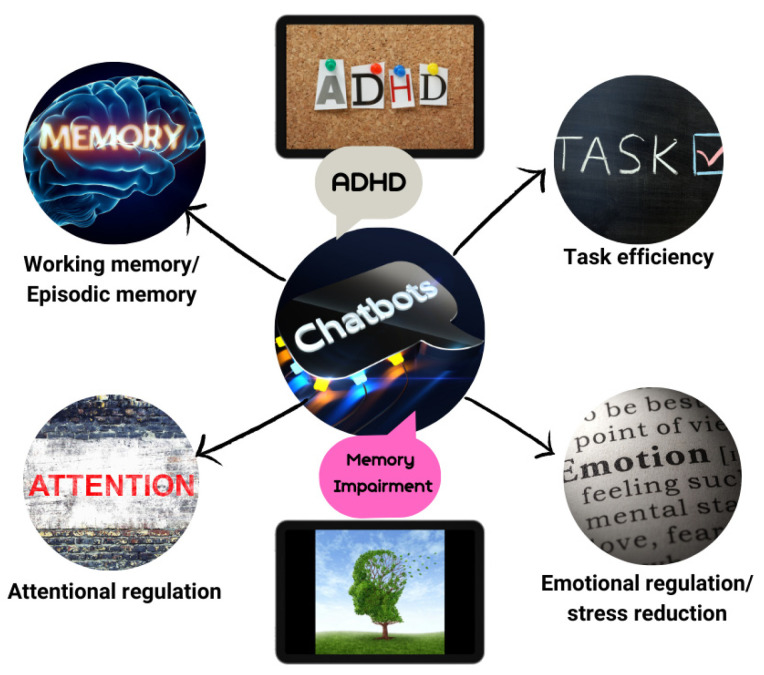
Chatbots and executive functions outcomes in special conditions.

Mauriello et al. 2021 [27] addressed the emotional regulation of EFs presenting significant stress reduction, indirectly supporting executive functions like emotional regulation and focus, with the users reporting better daily task management and reduced anxiety. Klarin et al., 2024 [14], in parity with the other studies had findings supporting the increase in in-task efficiency and working memory through chatbot-assisted schoolwork. The study suggested that chatbots helped adolescents’ structure and complete assignments with minimal cognitive strain. Fabio et al., 2024 [31] found enhanced cognitive flexibility and reasoning skills through the use of chatbot prompts that encouraged participants to explore diverse solutions and justify their reasoning, improving complex problem-solving. Koivisto and Grassini, 2023 [29], discovered data that supported the improvement of creativity and divergent thinking, which are considered to be key aspects of cognitive flexibility. Participants seemed to generate more novel and original ideas during problem-solving tasks compared to baseline. Better task planning and behavioral regulation, particularly in children with ADHD, as well as reduction in hyperactivity and improved adherence to structured task frameworks, were observed by [26]. Kim et al., 2024 [63] results suggested significant improvement in episodic memory and reduced anxiety levels, while participants presented fewer memory errors and improved emotional regulation, contributing to better daily self-management. Jang et al., 2021 [21] findings also implied a reduction in impulsivity and improved attention in adults with ADHD; also, emotional regulation improved significantly, enabling participants to better manage daily routines. Sun et al., 2023 [25] found improved attention and behavioral control in children with ADHD. Chatbot-driven exercises targeted cognitive domains like inhibition and memory, leading to better focus and self-control. In Rostami and Abadi, 2023 [22], a study discovered mixed results on working memory. Some participants showed improvement during chatbot-assisted tasks, while others experienced challenges due to over-reliance on AI for cognitive support. Finally, Chou and Hsu, 2021 [30] showcased results affecting cognitive load, improving task efficiency and mental clarity. Participants found chatbot interactions streamlined decision-making processes and reduced perceived mental effort (Figure 6).

### 4.3. RQ3. What Was Their Duration Effect?

Klarin et al., 2024 [14] showed that task efficiency and working memory improvements were short-term, lasting only during chatbot-assisted assignments. In Fabio et al., 2024 [31], gains in cognitive flexibility and reasoning skills were task-specific and limited to the duration of chatbot interactions. In addition, in Koivisto and Grassini, 2023 [29], the improvements in divergent thinking, creativity, and divergent thinking were observed only during the task performance with chatbots. Behavioral regulation and task adherence improvements were sustained throughout the six-week intervention, but no post-intervention follow-up was reported in [26] study. In a study by Kim et al., 2024 [28] about episodic memory, improvements and reduced anxiety were sustained for up to one month post-intervention, indicating medium-term retention of effects. Jang et al., 2021 [21] reduced impulsivity, improved attention, and emotional regulation were maintained for one month post-intervention, indicating significant short-term retention. In Sun et al., 2023 [25] study, attention and behavioral control improvements were evident during the four-week intervention; on the contrary, long-term impacts were not measured or reported. In a study by Rostami and Abadi, 2023 [22], working memory improvements were also limited to the immediate task duration, presenting no evidence of long-term retention or transfer of benefits. Finally, according to Chou and Hsu, 2021 [30], cognitive load reduction occurred immediately during interactions and was limited to the active use of the chatbot.

## 5. Discussion

Integrating AI systems like chatbots into various domains, including education and therapy, can be characterized as transformative. Executive functions can encompass a wide range of higher mental abilities such as working memory, attention, cognitive flexibility, and emotional-inhibitory control, leading to specific and goal-oriented behaviors and actions. This set of abilities every human possesses is tightly associated with learning adaptability and development in dynamic environmental stages [9]. On the other hand, AI technologies can express several of these skills by emulating problem-solving, reasoning, and planning capabilities that align well with these processes, finding it helpful to contribute to several conditions that seem to be affected by EF irregularities like ADHD, TBI, autism, and Alzheimer’s [21,25,26,67]. We should also raise concerns based on previous studies that AI chatbots in the context of Education 4.0 [68,69,70] emerge as pivotal tools leading a paradigm shift facilitating interaction autonomy and immediate feedback, enhancing the cognitive and emotional engagement of students, and leading to the promotion of learning needs fostering inclusion and equity [21,24]. Nonetheless, despite the benefits of AI, several negative elements can impact the user’s experience, leading to unwanted effects [71]. Some of them include the overreliance on technology, which in this case the users may become overly dependent on their use, influencing the development of critical thinking and problem-solving. Another downside can be the reduction in human interaction as chatbots may interfere with the relations and interactions between the instructor and the learner [72,73]. Other implications can relate to several subjects that chatbots and LLMs, in general, can present as defects. Algorithmic bias and hallucinations, or inaccurate and misleading information, are the main reasons that can negatively affect the users’ experience [72,73,74]. More specifically, AI systems can be as good as the data that they are trained on, so if the training data may contain several biases, the chatbots would respond adequately [75]. The findings of this systematic review highlight the significance and the impact as well as the potential of conversational AI chatbots in targeting and improving highly cognitive functions like executive ones, establishing gains in hot and cold components. The results of this study derived from a diverse set of different studies that performed various types of methodologies, study designs, and interventions utilizing numerous variations in this technology, showing improvements to several conditions that impact EFs, driving better memory recall, stress management, and better academic support [22,26,27,31].

The majority of the studies yielded positive effects among samples, underlining the flexibility and effectiveness in their use related to EFs. More specifically, chatbots that were used, like Tobaki [21], NUROW [25], and ForME [26], demonstrated significant improvement in emotional and behavioral regulation and control, attention, and task planning, especially in the populations that were tested (ADHD). Chatbots produced with the use of ChatGPT [14,28,29] improved working memory and task efficiency, cognitive flexibility and reasoning, as well as creativity and divergent thinking, with only one study referring to mixed results in working memory and risk of over-reliance. Other specified chatbots, like Popbots [27] and cognitive training chatbots [28], produced stress reduction and positive episodic memory effects for the participants as well as better emotional regulation. The populations tested were related mostly to a specific neurodevelopmental disorder (ADHD) [21,25,26], age-related memory impairment [28], and the general population [14,22,27,29,30,31].

Many of the studies presented several limitations, especially related to the duration of the effects, as most of them presented immediate effects and did not include any follow-ups. Only two studies [21,28] presented 1 month post-intervention positive effects. Other limitations that were discovered were the small sample sizes, lack of diverse populations [21,25,27,28] and, besides the diversity of settings in which these chatbots were tested and intervened (home, educational settings, clinics), they were performed in well-controlled settings and conditions that did not generalize in real-world environments and situations.

These limitations yield the need to produce higher-quality studies that observe not only the short-term effects of the chatbots but also the long-term impact they present on EFs. Also, a need for a unified framework is needed to be able to reproduce specific and guided evidence-based practices, specific prompts, and uses of these chatbots in order to improve long-term the effectiveness of executive functioning skills among different types of populations that suffer from the reduction in their functional capacity. Last but not least, it is crucial to assess the technological and ethical ramifications of AI systems and make sure they are used responsibly and openly. Chatbots and AI systems should be used to supplement human knowledge, discretion, and creativity, not to replace it. As we stated previously, it is crucial for researchers to rigorously assess and confirm the information offered by chatbots before utilizing them in their work [54]. The ethical implications and the lack of proper educational use may affect the participants for several reasons, including data privacy, algorithmic bias, and hallucinations [72,73,74,75] (Table 2).

## 6. Conclusions

To conclude, this study is the first to our knowledge that highlights the potential and benefits of AI conversational chatbots as therapeutic agents specifically to improve executive functions. Despite the positive effects, the findings reported several implications due to the lack of a diverse population, small sample size, missing longitudinal studies, and small duration of effects. In future research, this type of technology can be used in different contexts and used by a variety of educators or specialists, such as therapists (occupational therapists, speech and language therapists, physiotherapists, and psychologists) as well as parents and caregivers to support the needs of individuals of different age groups and conditions studying the long-term effects of their applications. In our study, we observed the effects of their use in a variety of contexts (home-based, educational settings, and special gamified settings), showcasing the need for them to be applied mostly in real-life situations and conditions. Last but not least, our observations also highlighted the need for a unified framework for future study protocols that may refer specifically to how chatbots can be prompted to maximize the efficiency of EFs in different age groups and conditions, as well as address all the potential ethical, threatening, and practical considerations about their use.

## Figures and Tables

**Table 1:** Central search strings.

Central Search Strings
“AI chatbots” OR “chatbots” OR “conversational agents” AND “executive functions” OR “attention” OR “memory” OR “cognitive load” OR “emotional regulation” AND “AI chatbots” AND (“executive functions” OR “attention” OR “memory” OR “cognitive load” OR “emotional regulation”) AND “Conversational agents” AND (“executive functions” OR “attention” OR “memory” OR “cognitive load” OR “emotional regulation”) AND “chatbots” AND (“executive functions” OR “attention” OR “memory” OR “cognitive load” OR “emotional regulation”)

**Table 2:** Summary of the included studies for analysis.

Studies	Study Design	Country	Population/Sample Size	Condition	Context	Intervention/Chatbot Type	Outcome Measures	Key Findings	Duration Effects	Limitations
Klarin et al., 2024 [14]	Cross-sectional survey	Sweden	*n* = 744 adolescents (12–19; 46% female overall)	General Population	Not specified	Generative AI chatbot (ChatGPT)	BRIEF-2 (executive function), grades	Improved task efficiency and working memory	Immediate; task-specific effects	Risk of over-reliance on chatbots
Jang et al., 2021 [21]	Pilot RCT	Republic of Korea	*n* = 46 adults (19–60; 56% female)	ADHD	Home-based	Psychoeducational chatbot (Tobaki chatbot)	CAARS (ADHD symptoms), SAS, QIDS-SR	Reduced ADHD symptoms, improved attention	1 month post-intervention	Limited follow-up
Rostami And Abadi, 2023 [22]	Single-subject AB design	UK	*n* = 3 students (11–13; gender not specified)	General Population	classroom	Generative AI chatbot (ChatGPT chatbot)	N-Back test (working memory)	Mixed results in working memory; risk of over-reliance	Immediate; no follow-up	Small sample size; limited generalization
Sun et al., 2023 [25]	Feasibility study	Republic of Korea	*n* = 27 children (6–12; 82% male)	ADHD	Gamified therapy setting	Gamified cognitive chatbot NUROW gamified cognitive chatbot)	ARS, CBCL	Improved attention and behavioral regulation	Immediate; during intervention	No long-term evaluation
Park et al., 2024 [26]	Randomized controlled trial (RCT)	Republic of Korea	*n* = 132 children (6–7; 51% male)	Children with ADHD	Elementary school	Goal-directed chatbot (ForME chatbot)	ADHD RS, BRIEF	Improved behavioral regulation and task planning	During 6-week intervention; no follow-up	Lack of post-intervention monitoring
Mauriello et al. 2021 [27]	Mixed-methods exploratory study	USA	*n* = 47 adults (18–74; 57% female)	General population	University	Stress management chatbot (Popbots)	PHQ-4 (stress levels)	Stress reduction and better emotional regulation	Immediate; no sustained effects	No long-term evaluation
Kim et al., 2024 [28]	Longitudinal intervention study	USA	*n* = 32 older adults (60+; 87% female)	Memory impairment	Home-based	Cognitive training chatbot	CANTAB (memory), GDS, GAI	Episodic memory improvement, anxiety reduction	1 month post-intervention	Small sample size
Koivisto and Grassini, 2023 [29]	Experimental comparative study	UK, USA, Canada	*n* = 256 adults (19–40; 42% female)	General Population	Not specified	Generative AI chatbot (ChatGPT 4, copy.ai)	Semantic distance, creativity ratings	Improved creativity and divergent thinking	Task-specific; immediate	Limited context for real-world application
Chou and Hsu, 2021 [30]	SEM analysis	Not Specified	*n* = 193 chatbot users (gender not specified)	General Population	online	Service-oriented chatbot (cognitive load reduction chatbot)	Cognitive load, user attitudes	Reduced cognitive load, improved task clarity	Immediate; limited to interactions	Context-specific findings
Fabio et al., 2024 [31]	Exploratory study	Not specified	*n* = 126 university students (18–32; 59% female)	General Population	Academic settings	General-purpose chatbot (ChatGPT chatbot)	Cognitive reasoning tests	Enhanced cognitive flexibility and reasoning	Task-specific; immediate	Limited generalizability of findings
